# Supplementary material for: It takes more than a machine: A pilot feasibility study of point-of-care HIV-1 viral load testing at a lower-level health center in rural western Uganda
Source: PLOS Glob Public Health. 2023 Mar 27;3(3):e0001678. doi: 10.1371/journal.pgph.0001678 (PMC10042348; doi:10.1371/journal.pgph.0001678)
Supplement: S1 File — (PDF) [file pgph.0001678.s001.pdf]

1. ELIGIBILITY ASSESSMENT

Record ID ________________________

A. Xpert Study Eligibility Assessment

Screen Date ________________________

Age ≥ 18 years
◯ Yes
◯ No

Receives routine HIV/ART care at Bugoye Health Centre?
◯ Yes
◯ No

Completed informed consent?
◯ Yes
◯ No

ELIGIBLE

NOT ELIGIBLE

B. Distance-to-Clinic Study

Screen Date ________________________

Eligible for Xpert study (above)?
◯ Yes
◯ No

Completed informed consent?
◯ Yes
◯ No

ELIGIBLE

NOT ELIGIBLE

2A. INTAKE VISIT - Demographics

Record ID	______________________
Research Assistant entering data	◯ Ngelese Herbert<br>◯ Ronnie Ndizeye<br>◯ Other
If Other, please type name here:	______________________
Study ID	______________________<br>(Date - First Two Letters of Village - First Letter Name - Number for that day. Do not use dashes. For example, if a patient named Ross Boyce from Izinga was the 4th patient enrolled on August 7th, the ID would be 07IZR004)
ART Clinic ID	______________________<br>(ID used for routine care at BHC)
What is your gender?	◯ Male<br>◯ Female
What is your preferred religion?	◯ Protestant<br>◯ Catholic<br>◯ Pentecostal<br>◯ Muslim<br>◯ Other
Which other religion?	______________________
What is your marital status?	◯ Not married<br>◯ Married<br>◯ Domestic partnership<br>◯ Separated<br>◯ Divorced<br>◯ Widowed
Do you have children?	◯ Yes<br>◯ No
How many children do you have?	◯ 1<br>◯ 2<br>◯ 3<br>◯ 4<br>◯ 5<br>◯ 6<br>◯ 7<br>◯ 8<br>◯ 9<br>◯ ≥10

How old is your oldest child?	______________________ (Years)
How old is your youngest child?	______________________ (Years)
Do any of your children have HIV?	◯ Yes<br>◯ No
Are you currently pregnant?	◯ Yes<br>◯ No
What is the highest level of education you have completed?	◯ No schooling<br>◯ Primary<br>◯ Secondary<br>◯ Tertiary
What is your age?	______________________
Is the exact date of birth known?	◯ Yes<br>◯ No
What is your date of birth?	______________________
Demographics verified with ID card?	◯ Yes<br>◯ No
What type of ID card?	◯ National ID Card<br>◯ Driving permit<br>◯ Passport<br>◯ Birth Certificate
Intake and Demographics Form Comments	______________________ (Record any comments or issues regarding the form above here.)

2B. INTAKE VISIT - Village of Residence

Record ID ____________________

Village Information

Does the participant live in Bugoye Sub-County

◯ Yes
◯ No

Parish of Residence

◯ Bugoye
◯ Ibanda
◯ Katooke
◯ Kibirizi
◯ Muhambo
(select one)

What village do you live in? (Bugoye Parish only)

◯ Bugoye
◯ Kanyanamigho
◯ Kisamba 1
◯ Kisamba 2
◯ Muramba 1
◯ Muramba 2
◯ Rwakingi 1A
◯ Rwakingi 1B
(select one)

What village do you live in? (Ibanda Parish only)

◯ Ibanda 1
◯ Ibanda 2
◯ Nyakabugha
◯ Kiharara
◯ Mihunga
◯ Mirimbo
◯ Nyakalingijo
◯ Ruboni
(select one)

What village do you live in? (Katooke Parish only)

◯ Katooke 1
◯ Katooke 2
◯ Kemihoko
◯ Kihindi
◯ Kinyangoye
◯ Kirongo
◯ Mapata
◯ Muleheya
◯ Nyangonge
(select one)

What village do you live in? (Kibirizi Parish only)

◯ Bulindiguru
◯ Ihani
◯ Kasanzi
◯ Kibirizi
◯ Kikokera
(select one)

What village do you live in? (Muhambo Parish only)	◯ Bunyangoni<br>◯ Katumba<br>◯ Maghoma<br>◯ Muhambo<br>◯ Nduguthu East<br>◯ Nduguthu West<br>(select one)
Who is your VHT? (Bugoye Parish only)	◯ Mumbere Dorothy (Bugoye)<br>◯ Tinka Tadeo (Bugoye)<br>◯ Babona Gertrude (Bugoye)<br>◯ Nyabutundu Zeresi (Bugoye)<br>◯ Magezi Paul (Bugoye)<br>◯ Mibiri Catherine (Kanyanamigho)<br>◯ Mugisha Adrian (Kanyanamigho)<br>◯ Sunday Leo (Kanyanamigho)<br>◯ Businge Paul (Kanyanmigho)<br>◯ Edreda Bagenda (Kanyanamigho)<br>◯ Musoki Delphina (Kisamba 1)<br>◯ Muhindo Jonan (Kisamba 1)<br>◯ Mibusa Erisania (Kisamba 1)<br>◯ Isingoma Yeremiah (Kisamba 1)<br>◯ Muhindo Medius (Kisamba 1)<br>◯ Bwambale Maliseri (Kisamba 2)<br>◯ Kabugho Alice (Kisamba 2)<br>◯ Kabanga James (Kisamba 2)<br>◯ Biira Eliza (Kisamba 2)<br>◯ Biira Evanis (Kisamba 2)<br>◯ Tusiime Scovia (Muramba 1)<br>◯ Odjan Jonathan (Muramba 1)<br>◯ Musoki Peregerina (Muramba 1)<br>◯ Mukuke Esther (Muramba 1)<br>◯ Masereka Samson (Muramba 1)<br>◯ Musoki Peskezia (Muramba 1)<br>◯ Musoki Esther (Muramba 2)<br>◯ Warufu Ibrahim (Muramba 2)<br>◯ Tsongo Doreen (Muramba 2)<br>◯ Mbindule Agnes (Muramba 2)<br>◯ Mwamini Munoli (Rwakingi 1A)<br>◯ Nsabebera Peter (Rwakingi 1A)<br>◯ Katusabe Florence (Rwakingi 1A)<br>◯ Tuhirirwe Cleophas (Rwakingi 1A)<br>◯ Kobusinge Slyvia (Rwakingi 1B)<br>◯ Mbonagaija Nestor (Rwakingi 1B)<br>◯ Muculezi Florence (Rwakingi 1B)<br>◯ Katalibaho Baturumayo (Rwakingi 1B)<br>◯ Biira Justine (Rwakingi 1B)<br>(select one)

Who is your VHT? (Ibanda Parish only)

◯ Kabugho Justine (Ibanda 1)
◯ Muthahunga Pelucy (Ibanda 1)
◯ Kabugho Babra (Ibanda 1)
◯ Biira Faith (Ibanda 1)
◯ Masa Jeremiah (Ibanda 1)
◯ Mbabazi Consolata (Ibanda 2)
◯ Baluku Bosco (Ibanda 2)
◯ Musoki Mary (Ibanda 2)
◯ Kusemererwa Jesca (Ibanda 2)
◯ Biira Grace (Ibanda 2)
◯ Biira Catherine (Nyakabugha)
◯ Muhindo Boniface (Nyakabugha)
◯ Kule John Kapalaya (Nyakabugha)
◯ Wangai Zeresi (Nyakabugha)
◯ Kabugho Moresi (Nyakabugha)
◯ Mbusa Asanasio (Kiharara)
◯ Baluku Jaruson (Kiharara)
◯ Bagenda Mary (Kiharara)
◯ Baluku Remegio (Kiharara)
◯ Musoki Joyline (Kiharara)
◯ Kahnido Rhoda (Kiharara)
◯ Musoki Roset (Mihunga)
◯ Masereka Jeremiah (Mihunga)
◯ Mateke Eliphaz (Mihunga)
◯ Kule Solomon (Mihunga)
◯ Kisembo Charles (Mihunga)
◯ Mukanirwa Josephat (Mirimbo)
◯ Masereka Solomon (Mirimbo)
◯ Kabugho Jones (Mirimbo)
◯ Masereka Ronald (Mirimbo)
◯ Tumusiime Jane (Mirimbo)
◯ Muhindo Joyline (Ruboni)
◯ Ithungu Splanza (Ruboni)
◯ Musoki Agnes (Ruboni)
◯ Bwambale Samwel (Ruboni)
◯ Biira Marion (Ruboni)
(select one)

Who is your VHT? (Katooke Parish only)

◯ Masika Jonesi (Katooke 1)
◯ Biira Salome (Katooke 1)
◯ Biira Miria (Katooke 1)
◯ Fundi Jonany (Katooke 1)
◯ Baluku Milton (Katooke 1)
◯ Bwambale Jackson (Katooke 2)
◯ Katya Pulikeria (Katooke 2)
◯ Biira Evaket (Katooke 2)
◯ Thabughakibi Sarapio (Katooke 2)
◯ Kabugho Agnes (Katooke 2)
◯ Nyamihanda Petronila (Kemihoko)
◯ Sunday Emmanuel (Kemihoko)
◯ Kamani Bernada (Kemihoko)
◯ Muhindo Amon (Kemihoko)
◯ Akolebirungi Robina (Kemihoko)
◯ Kathasibwe Isaac (Kihindi)
◯ Sukulhubana Sanasio (Kihindi)
◯ Janet Kabanda (Kihindi)
◯ Masika Slyvia (Kihindi)
◯ Kabau Janackaison (Kihindi)
◯ Biira Janet (Kihindi)
◯ Mubanami Kule Jackson (Kinyangoye)
◯ Katasibwe Johnson (Kinyangoye)
◯ Mukanirwa Wilson (Kinyangoye)
◯ Musoki Rahabu (Kinyangoye)
◯ Kiiza Evelyn (Kinyangoye)
◯ Ithungu Agnes (Kirongo)
◯ Thungu Alice (Kirongo)
◯ Ndungu Alfose (Kirongo)
◯ Kisoke Janet (Kirongo)
◯ Bwambale Ronald (Kirongo)
◯ Biira Eddy (Mapata)
◯ Bitswande Rabson (Mapata)
◯ Kithanda Harriet (Mapata)
◯ Agnes Ndungu (Mapata)
◯ Biira Scovia (Mapata)
◯ Masereka Richard (Mulehe)
◯ Musumba Jocknus (Mulehe)
◯ Baluku Neckson (Mulehe)
◯ Sadress Kalwana (Mulehe)
◯ Kabugho Betina (Mulehe)
◯ Ngunule James (Nyangonge)
◯ Ngunule Lydia (Nyangonge)
◯ Kyegheka Yosufu (Nyangonge)
◯ Jusi Jeremiah (Nyangonge)
◯ Banyamire Jocknus (Nyangonge)
(select one)

Who is your VHT? (Kibirizi Parish only)	◯ Rukundo Lilian (Bulindiguru)<br>◯ Sibaya Johnson Bisusi (Bulindiguru)<br>◯ Biira Harriet (Bulindiguru)<br>◯ Kathabana Esther (Bulindiguru)<br>◯ Muthaghanza Josia (Bulindiguru)<br>◯ Muhindo Michael (Ihani)<br>◯ Thembo Zepher (Ihani)<br>◯ Ithungu Rachael (Ihani)<br>◯ Kabugho Harriet (Ihani)<br>◯ Asimwe Yeresi (Ihani)<br>◯ Tibulihwa Robert (Kasanzi)<br>◯ Muhindo Jennifer (Kasanzi)<br>◯ Mbabazi Mackline (Kasanzi)<br>◯ Muhindo Apophia (Kasanzi)<br>◯ Mwiraghulu Sele (Kasazni)<br>◯ Kisumbusa Esther (Kibirizi)<br>◯ Musubaho William (Kibirizi)<br>◯ Mathina Edison (Kibirizi)<br>◯ Muhindo Annet (Kibirizi)<br>◯ Mbambu Jolly (Kibirizi)<br>◯ Aida Bwambale (Kikokera)<br>◯ Bwambale Zalimon (Kikokera)<br>◯ Jane Kipura (Kikokera)<br>◯ Aida Kule (Kikokera)<br>(select one)
Who is your VHT? (Muhambo Parish only)	◯ Kachingwe Agnes (Bunyangoni)<br>◯ Ngotho Longina (Bunyangoni)<br>◯ Mwirirya Zepher (Bunyangoni)<br>◯ Erion Ngunule (Bunyangoni)<br>◯ Yayeri Mbasa (Bunyangoni)<br>◯ Juliet Mumbere (Katumba)<br>◯ Bwambale Baker Edson (Katumba)<br>◯ Bathulwamo Jonathan (Katumba)<br>◯ Biira Felestus (Katumba)<br>◯ Biira Doreen (Katumba)<br>◯ Ndathu Selevano (Maghoma)<br>◯ Mbambu Kevin (Maghoma)<br>◯ Masereka Michael (Maghoma)<br>◯ Mwirirya Zephanus (Maghoma)<br>◯ Muhindo Selevano (Maghoma)<br>◯ Mukanirwa Emmy (Muhambo)<br>◯ Betty Kasimba (Muhambo)<br>◯ Kighoma Moris (Muhambo)<br>◯ Kipura John (Muhambo)<br>◯ Nyoro Yeresi (Muhambo)<br>◯ Bukwirwa Rose (Nduguthu East)<br>◯ Kyakurugaha Clovis (Nduguthu East)<br>◯ Kisembo Margaret (Nduguthu East)<br>◯ Awori Eunice (Nduguthu East)<br>◯ Masereka Girisoni (Nduguthu East)<br>◯ Businge Amon (Nduguthu West)<br>◯ Tuhaise Joan (Nduguthu West)<br>◯ Bwambale Mathondi Eric (Nduguthu West)<br>◯ Byaruhanga Joseph (Nduguthu West)<br>◯ Munoli Agnes (Nduguthu West)<br>(select one)
If the participant does not live in Bugoye Sub-County, please list the sub-county of residence	__________________________
If the participant does not live in Bugoye Sub-County, please list the parish of residence	__________________________

If the participant does not live in Bugoye Sub-County, please list the village of residence	__________________________________
Comments	____

2C. INTAKE VISIT - ART Adherence

Record ID ______________________________

COMPLETE THIS SECTION ONLY IF THE PATIENT HAS BEEN ON ART PRIOR TO TODAY'S VISIT.

"Many people find it difficult to always take all of their tablets. Some people become busy and forget to carry their tablets with them. It is important for the study that we understand how participants are really doing with taking their tablets. I would now like to ask you a few questions about how you have been taking your tablets since last month. You will still be able to participate in the study if you missed some of your tablets."

Please tell me your ability to take all of your ART medicines as directed in the past month.

- ◯ Very poor
- ◯ Poor
- ◯ Fair
- ◯ Good
- ◯ Very good
- ◯ Excellent

How often did you take all of your ART tablets in the past month?

- ◯ None of the time
- ◯ A little of the time
- ◯ Some of the time
- ◯ A good bit of the time
- ◯ Most of the time
- ◯ All of the time

What percent of the time were you able to take your tablets exactly as directed in the past month?

(For example, 0% means you took none of the ART tablets, 50% means you took half of them, and 100% means you took all of them exactly as directed.)

- ◯ 0%
- ◯ 10%
- ◯ 20%
- ◯ 30%
- ◯ 40%
- ◯ 50%
- ◯ 60%
- ◯ 70%
- ◯ 80%
- ◯ 90%
- ◯ 100%

During the past 4 days, on how many days have you missed taking all of your doses?

- ◯ None
- ◯ One day
- ◯ Two days
- ◯ Three days
- ◯ Four days

Most ART medications need to be taken on a schedule, such as "every night," "every morning," or "2 time a day." How closely did you follow your specific schedule over the last four days?

- ◯ Never
- ◯ Some of the time
- ◯ About half of the time
- ◯ Most of the time
- ◯ All of the time

Do any of your ART medications have special instructions, such as "take with food" or "on an empty stomach" or "with plenty of fluids"?

- ◯ Yes
- ◯ No

How often did you follow those special instructions over the last four days?	◯ Never<br>◯ Some of the time<br>◯ About half of the time<br>◯ Most of the time<br>◯ All of the time
Some people find that they forget to take their pills on the weekend days. Did you miss any of your medications last weekend - last Saturday or Sunday?	◯ Yes<br>◯ No
When was the last time you missed any of your medications?	◯ Within the past week<br>◯ 1-2 weeks ago<br>◯ 2-4 weeks ago<br>◯ 1-3 months ago<br>◯ More than 3 months ago<br>◯ Never skip medications

"People may miss taking their medications for various reasons. Here is a list of possible reasons why you may miss taking your medications. How often have you missed taking your ART medications because you:"

Ran out of pills?	◯ Never<br>◯ Rarely<br>◯ Sometimes<br>◯ Often
Wanted to avoid side effects?	◯ Never<br>◯ Rarely<br>◯ Sometimes<br>◯ Often
Did not want others to notice you taking medication?	◯ Never<br>◯ Rarely<br>◯ Sometimes<br>◯ Often
Felt like the drug was toxic/harmful?	◯ Never<br>◯ Rarely<br>◯ Sometimes<br>◯ Often
Did the participant receive adherence counseling at today's clinic visit?	◯ Yes<br>◯ No
Adherence Comments	______________________________<br>(Record any comments or issues regarding the form above here.)

2D. INTAKE VISIT - Concurrent Medications

Record ID	______________________
Ask the participant and review the chart.<br><br>In the past three days, have you taken any of the following medications? Check all that apply.	
Vitamins	☐ Multivitamin<br>☐ Calcium carbonate<br>☐ Magnesium oxide
TB treatment	☐ Rifampin<br>☐ Isoniazid<br>☐ Ethambutol<br>☐ Pyrazinamide<br>☐ Pyridoxine
Antiepileptics	☐ Carbamazapine<br>☐ Oxcarbazepine<br>☐ Phenobarbital<br>☐ Phenytoin<br>☐ Midazolam<br>☐ Triazolam<br>☐ Pimozide
Antidepressants	☐ Sertraline<br>☐ Fluoxetine<br>☐ Paroxetine<br>☐ Bupropion<br>☐ Trazodone<br>☐ Amitriptyline
Contraceptives	☐ OCPs<br>☐ DMPA (Depo-Provera)<br>☐ Implanon
Antidiabetics	☐ Metformin
Antibiotics	☐ Septrin<br>☐ Fluconazole<br>☐ Ciprofloxacin<br>☐ Acycolovir<br>☐ Azithromycin
Other	☐ Ergotamine<br>☐ St. John's Wort<br>☐ Probenicid
In the past three days, have you taken any other medications?	◯ Yes<br>◯ No
Which other medications	______________________

In the past three days, have you taken any traditional medications?	◯ Yes<br>◯ No
What type(s) of traditional medications?	__________________________
Medications Comments	__________________________<br>(Record any comments or issues regarding the form above here.)

2E. INTAKE VISIT - Visit Summary

Record ID ______________________

A. Administrative Summary

Visit Date ______________________

What time did the participant arrive at clinic today? ______________________

What is the current study phase?
- ◯ Phase 1 - Routine Testing
- ◯ Phase 2 - Phase 2 - GeneXpert testing available

B. Clinical Summary

Did the clinician recommend a change in the ART regimen today?
- ◯ Yes
- ◯ No

What is the new ART regimen that was started today?
- ◯ ABC/3TC/ATV
- ◯ AZT/3TC/ATV
- ◯ AZT/3TC/EFV
- ◯ AZT/3TC/NVP
- ◯ TDF/3TC/ATV
- ◯ TDF/3TC/DTG
- ◯ TDF//3TC/EFV
- ◯ TDF/3TC/LOP/r
- ◯ Other

(3TC = lamivudine; ABC = abacavir; ATV = atazanavir, DTG = dolutegravir, EFV = efavirenz; LOP/r = lopinavir/ritonavir; NVP = nevirapine; TDF = tenofovir)

What other ART regimen (check all that apply)
- ☐ 3TC
- ☐ FTC
- ☐ AZT
- ☐ TDF
- ☐ ABC
- ☐ EFV
- ☐ NVP
- ☐ DTG
- ☐ ATV/r

What was the reason for the change in ART regimen?
- ◯ Failure due to resistance
- ◯ Side effects
- ◯ Other

If side effects were the reason for stopping, what were the side effects? ______________________

Other reason for the ART regimen change? ______________________

Did the participant receive Intensive Adherence Counseling (IAC) today?	◯ Yes<br>◯ No
What was the reason for IAC?	◯ Viral Load >1,000 copies<br>◯ Other
Other reason for IAC?	__________________________________
Was an Opportunistic Infection (OI) diagnosed today?	◯ Yes<br>◯ No
Which OI was diagnosed today? (check all that apply)	☐ Pulmonary tuberculosis<br>☐ Extrapulmonary tuberculosis<br>☐ Cryptococcal meningitis<br>☐ Pneumonia<br>☐ Kaposi sarcoma<br>☐ Esophagitis<br>☐ Other
What other OI was diagnosed today?	__________________________________
Were any other infections such as malaria, hepatitis, or sexually transmitted infections diagnosed today?	◯ Yes<br>◯ No
Which other infection was diagnosed today? (check all that apply)	☐ Malaria<br>☐ Hepatitis B<br>☐ Hepatitis C<br>☐ Syphilis<br>☐ Urethritis (male)<br>☐ Urethritis / Vaginal Discharge (female)<br>☐ Other
What other infection was diagnosed today?	__________________________________

C. Laboratory Summary

Did the clinician recommend CD4 testing today?	◯ Yes<br>◯ No
Why was CD4 testing recommended today?	◯ New diagnosis or initiating ART<br>◯ Viral Load >1,000 copies or Stage 3/4 Disease<br>◯ On treatment or prophylaxis for Cryptococcal disease<br>◯ Other
Other reason for CD4 testing	__________________________________
Did the clinician recommend viral load testing today?	◯ Yes<br>◯ No

Why was viral load testing recommended today?	◯ Started ART within the last 6 months<br>◯ Due for annual testing<br>◯ Viral Load >1,000 copies<br>◯ Pregnancy<br>◯ Other
Other reason for Viral Load testing	__________________________
How did the participant choose to receive his/her viral load results?	◯ Receive results at next scheduled visit<br>◯ By phone call when test results are available<br>◯ Wait for results today (PHASE 2 ONLY)<br>◯ Other
What was the other method of receiving viral load results?	__________________________
Did the patient stay to have blood drawn for testing?	◯ Yes<br>◯ No

D. CD4 Results - Only if ordered

What was the result of CD4 testing?	_____________________<br>(cells/mm3)
When was the CD4 test completed?	_____________________
When did the participant receive the CD4 test result?	_____________________
Were any other changes made to the treatment regimen or follow-up plan as a result of the CD4 results? Be sure to ask both the participant and the clinician.	◯ Yes<br>◯ No
Please document any changes made:	__________________________

E. Routine Viral Load Results - Only if ordered

What was the result of the ROUTINE viral load test?	◯ Not detectable<br>◯ < 1,000 copies<br>◯ ≥1,000 copies<br>(Routine = test sent to Central Public Health Lab)
Enter exact value of ROUTINE viral load test result?	_____________________
What date was the ROUTINE viral load test performed at the Central Public Health Lab?	_____________________
What date was the ROUTINE viral load test result received at BHC?	_____________________

What date was the ROUTINE viral load test result reported to the participant?	__________________________________
How did the participant actually receive his/her ROUTINE viral load results?	◯ At next visit<br>◯ By phone call<br>◯ Other
Describe other method of receiving ROUTINE viral load results	____
Were any other changes made to the treatment regimen or follow-up plan as a result of the ROUTINE viral load results? Be sure to ask both the participant and the clinician.	◯ Yes<br>◯ No
Please document any changes made:	____

F. Xpert Viral Load Results - Only if ordered (Phase 2 Only)

What was the result of the XPERT viral load test?	◯ Not detectable<br>◯ < 1,000 copies<br>◯ ≥1,000 copies
Enter exact value of XPERT viral load test result?	__________________________________
What date was the XPERT viral load test performed at BHC?	__________________________________
What date was the XPERT viral load test result reported to the participant?	__________________________________
If the patient chose to wait for the XPERT results today, did the participant actually receive their results today?	◯ Yes<br>◯ No
What time did the participant receive their XPERT results?	__________________________________
If the participant chose not to wait for results today, how did the participant actually receive his/her XPERT viral load results?	◯ At next visit<br>◯ By phone call<br>◯ Other
Describe other method of receiving XPERT viral load results	____
Did the participant receive Intensive Adherence Counseling today after receiving the XPERT viral load results?	◯ Yes<br>◯ No

Were any other changes made to the treatment regimen or follow-up plan as a result of the XPERT viral load results? Be sure to ask both the participant and the clinician.	◯ Yes<br>◯ No
Please document any changes made:	__________________________________

G. Follow Up Plan

Date of next scheduled appointment	___________________________
Comments	__________________________________<br>(Record any comments or issues regarding the form above here.)

3A. CHART REVIEW - HIV And ART History

Record ID ______________________

REVIEW THE CHART

Clinic enrollment date ______________________

Start date of ARTs ______________________

Has the participant been on ART for at least 1 year?
◯ Yes
◯ No

What ART regimen is the participant currently taking?
◯ ABC/3TC/ATV
◯ AZT/3TC/ATV
◯ AZT/3TC/EFV
◯ AZT/3TC/NVP
◯ TDF/3TC/ATV
◯ TDF/3TC/DTG
◯ TDF//3TC/EFV
◯ TDF/3TC/LOP/r
◯ Other
(3TC = lamivudine; ABC = abacavir; ATV = atazanavir, DTG = dolutegravir, EFV = efavirenz; LOP/r = lopinavir/ritonavir; NVP = nevirapine; TDF = tenofovir)

What other ART regimen? (Check all that apply.)
☐ 3TC
☐ FTC
☐ AZT
☐ TDF
☐ ABC
☐ EFV
☐ NVP
☐ DTG
☐ ATV/r

When did the patient start the current regimen? ______________________

Is this the participants first ART regimen?
◯ Yes
◯ No

What was the participants previous ART regimen before the current ART regimen?	◯ ABC/3TC/ATV<br>◯ AZT/3TC/ATV<br>◯ AZT/3TC/EFV<br>◯ AZT/3TC/NVP<br>◯ TDF/3TC/ATV<br>◯ TDF/3TC/DTG<br>◯ TDF//3TC/EFV<br>◯ TDF/3TC/LOP/r<br>◯ Other<br>(3TC = lamivudine; ABC = abacavir; ATV = atazanavir, DTG = dolutegravir, EFV = efavirenz; LOP/r = lopinavir/ritonavir; NVP = nevirapine; TDF = tenofovir)
What was the other ART regimen?	☐ 3TC<br>☐ FTC<br>☐ AZT<br>☐ TDF<br>☐ ABC<br>☐ EFV<br>☐ NVP<br>☐ DTG<br>☐ ATV/r
Start date of the prior ART regimen?	______________________________
Stop date of the prior ART regimen?	______________________________
Reason for regimen change?	◯ Failure due to resistance<br>◯ Side effects<br>◯ Other<br>◯ Unable to determine
Other reason for regimen change?	_____________________________________
Does the participant have a viral load result in the chart?	◯ Yes<br>◯ No
Date of most recent viral load	______________________________
Most recent viral load result	◯ Target not detected<br>◯ < 1,000 copies/mL<br>◯ >1,000 copies/mL
Please provide the exact value for the most recent viral load result	______________________________<br>(copies/mL)
Most recent CD4 count	______________________________
Date of most recent CD4 count	______________________________

Lowest CD4 count recorded in the chart?	______________________________
Date of lowest CD4 count recorded in the chart	______________________________
Has the participant ever been diagnosed with an Opportunistic Infection (OI)?	◯ Yes<br>◯ No
What Opportunistic Infections have been diagnosed in the past? (check all that apply)	☐ Pulmonary tuberculosis<br>☐ Extrapulmonary tuberculosis<br>☐ Cryptococcal meningitis<br>☐ Pneumonia<br>☐ Kaposi sarcoma<br>☐ Esophagitis<br>☐ Other
List any other Opportunistic Infections:	_____________________________________
Has the participant ever undergone Intensive Adherence Counseling (IAC)?	◯ Yes<br>◯ No
Date of most recent IAC	______________________________
HIV and ART History Comments	_____________________________________<br>(Record any comments or issues regarding the form above here.)

3B. CHART REVIEW - Laboratory Data

Record ID ____________________

REVIEW THE CHART.

RENAL

Date of most recent creatinine ____________________

Most recent creatinine result? ____________________
(mg/dL)

Please enter the unit for the creatinine result ____________________
(Ex. mg/dL, umol/L)

Date of most recent urinalysis ____________________

Most recent urine protein?
- ◯ Negative
- ◯ 1+
- ◯ 2+
- ◯ 3+
- ◯ 4+

Most recent urine specific gravity? ____________________

HEPATIC

Date of most recent liver function tests ____________________

Most recent AST result? ____________________

Please enter the unit for the AST result ____________________
(Ex. U/L)

Most recent ALT result? ____________________

Please enter the unit for the ALT result ____________________
(Ex. U/L)

Most recent alkaline phosphatase result? ____________________

Please enter the unit for the alkaline phosphatase result	______________________ (Ex. U/L)
Most recent total bilirubin result?	______________________
Please enter the unit for the total bilirubin result	______________________ (Ex. mg/dL, umol/L)
Date of most recent HBsAg test?	______________________
Result of most recent HBsAg test?	◯ Negative<br>◯ Positive

HEMATOLOGIC

Date of most recent hemoglobin test?	______________________
Result of most recent hemoglobin test?	______________________
Most recent WBC count?	______________________
Most recent platelet count?	______________________

INFECTIOUS DISEASE

Date of most recent sputum smear for TB?	______________________
Result of sputum smear for TB?	______________________
Date of most recent Gene Xpert test for TB?	______________________
Result of Gene Xpert test for TB?	◯ Negative<br>◯ Positive

PREGNANCY TESTS

Skip is participant is male.

Date of most recent urine pregnancy test? __________________________________

Result of urine pregnancy test?
- ◯ Negative
- ◯ Positive

Date of other pregnancy test __________________________________

What other type of pregnancy test? __________________________________

Result of other pregnancy test? __________________________________

Lab Results Comments ____
(Record any comments or issues regarding the form above here.)

4. RETURN VISIT 1 - Visit Summary

Record ID ______________________

A. Administrative Summary

Visit Date ______________________

What time did the participant arrive at clinic today? ______________________

What is the current study phase?
- ◯ Phase 1 - Routine Testing
- ◯ Phase 2 - Phase 2 - GeneXpert testing available

B. Clinical Summary

Did the clinician recommend a change in the ART regimen today?
- ◯ Yes
- ◯ No

What is the new ART regimen that was started today?
- ◯ ABC/3TC/ATV
- ◯ AZT/3TC/ATV
- ◯ AZT/3TC/EFV
- ◯ AZT/3TC/NVP
- ◯ TDF/3TC/ATV
- ◯ TDF/3TC/DTG
- ◯ TDF//3TC/EFV
- ◯ TDF/3TC/LOP/r
- ◯ Other

(3TC = lamivudine; ABC = abacavir; ATV = atazanavir, DTG = dolutegravir, EFV = efavirenz; LOP/r = lopinavir/ritonavir; NVP = nevirapine; TDF = tenofovir)

What other ART regimen (check all that apply)
- ☐ 3TC
- ☐ FTC
- ☐ AZT
- ☐ TDF
- ☐ ABC
- ☐ EFV
- ☐ NVP
- ☐ DTG
- ☐ ATV/r

What was the reason for the change in ART regimen?
- ◯ Failure due to resistance
- ◯ Side effects
- ◯ Other

If side effects were the reason for stopping, what were the side effects? ______________________

Other reason for the ART regimen change? ______________________

Did the participant receive Intensive Adherence Counseling (IAC) today?	◯ Yes<br>◯ No
What was the reason for IAC?	◯ Viral Load >1,000 copies<br>◯ Other
Other reason for IAC?	__________________________________
Was an Opportunistic Infection (OI) diagnosed today?	◯ Yes<br>◯ No
Which OI was diagnosed today? (check all that apply)	☐ Pulmonary tuberculosis<br>☐ Extrapulmonary tuberculosis<br>☐ Cryptococcal meningitis<br>☐ Pneumonia<br>☐ Kaposi sarcoma<br>☐ Esophagitis<br>☐ Other
What other OI was diagnosed today?	__________________________________
Were any other infections such as malaria, hepatitis, or sexually transmitted infections diagnosed today?	◯ Yes<br>◯ No
Which other infection was diagnosed today? (check all that apply)	☐ Malaria<br>☐ Hepatitis B<br>☐ Hepatitis C<br>☐ Syphilis<br>☐ Urethritis (male)<br>☐ Urethritis / Vaginal Discharge (female)<br>☐ Other
What other infection was diagnosed today?	__________________________________

C. Laboratory Summary

Did the clinician recommend CD4 testing today?	◯ Yes<br>◯ No
Why was CD4 testing recommended today?	◯ New diagnosis or initiating ART<br>◯ Viral Load >1,000 copies or Stage 3/4 Disease<br>◯ On treatment or prophylaxis for Cryptococcal disease<br>◯ Other
Other reason for CD4 testing	__________________________________
Did the clinician recommend viral load testing today?	◯ Yes<br>◯ No

Why was viral load testing recommended today?	◯ Started ART within the last 6 months<br>◯ Due for annual testing<br>◯ Viral Load >1,000 copies<br>◯ Pregnancy<br>◯ Other
Other reason for Viral Load testing	__________________________
How did the participant choose to receive his/her viral load results?	◯ Receive results at next scheduled visit<br>◯ By phone call when test results are available<br>◯ Wait for results today (PHASE 2 ONLY)<br>◯ Other
What was the other method of receiving viral load results?	__________________________
Did the patient stay to have blood drawn for testing?	◯ Yes<br>◯ No

D. CD4 Results - Only if ordered

What was the result of CD4 testing?	______________________<br>(cells/mm3)
When was the CD4 test completed?	______________________
When did the participant receive the CD4 test result?	______________________
Were any other changes made to the treatment regimen or follow-up plan as a result of the CD4 results? Be sure to ask both the participant and the clinician.	◯ Yes<br>◯ No
Please document any changes made:	__________________________

E. Routine Viral Load Results - Only if ordered

What was the result of the ROUTINE viral load test?	◯ Not detectable<br>◯ < 1,000 copies<br>◯ ≥1,000 copies<br>(Routine = test sent to Central Public Health Lab)
Enter exact value of ROUTINE viral load test result?	______________________
What date was the ROUTINE viral load test performed at the Central Public Health Lab?	______________________
What date was the ROUTINE viral load test result received at BHC?	______________________

What date was the ROUTINE viral load test result reported to the participant?	__________________
How did the participant actually receive his/her ROUTINE viral load results?	◯ At next visit<br>◯ By phone call<br>◯ Other
Describe other method of receiving ROUTINE viral load results	__________________
Were any other changes made to the treatment regimen or follow-up plan as a result of the ROUTINE viral load results? Be sure to ask both the participant and the clinician.	◯ Yes<br>◯ No
Please document any changes made:	__________________

F. Xpert Viral Load Results - Only if ordered (Phase 2 Only)

What was the result of the XPERT viral load test?	◯ Not detectable<br>◯ < 1,000 copies<br>◯ ≥1,000 copies
Enter exact value of XPERT viral load test result?	__________________
What date was the XPERT viral load test performed at BHC?	__________________
What date was the XPERT viral load test result reported to the participant?	__________________
If the patient chose to wait for the XPERT results today, did the participant actually receive their results today?	◯ Yes<br>◯ No
What time did the participant receive their XPERT results?	__________________
If the participant chose not to wait for results today, how did the participant actually receive his/her XPERT viral load results?	◯ At next visit<br>◯ By phone call<br>◯ Other
Describe other method of receiving XPERT viral load results	__________________
Did the participant receive Intensive Adherence Counseling today after receiving the XPERT viral load results?	◯ Yes<br>◯ No

Were any other changes made to the treatment regimen or follow-up plan as a result of the XPERT viral load results? Be sure to ask both the participant and the clinician.

○ Yes
○ No

Please document any changes made:

____

G. Follow Up Plan

Date of next scheduled appointment

____

Comments

____
(Record any comments or issues regarding the form above here.)
