# Supplementary material for: It takes more than a machine: A pilot feasibility study of point-of-care HIV-1 viral load testing at a lower-level health center in rural western Uganda
Source: PLOS Glob Public Health. 2023 Mar 27;3(3):e0001678. doi: 10.1371/journal.pgph.0001678 (PMC10042348; doi:10.1371/journal.pgph.0001678)
Supplement: Attachment — Submitted filename: PLOS_GPH_R2R (21DEC22).docx [file pgph.0001678.s002.docx]

December 27, 2022

Sabine Hermans, MD, PhD

Academic Editor

*PLOS Global Public Health*

Dear Dr. Hermans:

Thank you for the constructive review of our manuscript entitled, “It takes more than a machine: A pilot feasibility study of health systems capacity building for point-of-care HIV-1 viral load testing in rural western Uganda” (PGPH-D-22-01433). Please find our responses (in blue) to reviewers’ comments below.

**Journal Requirements:**

**Comment 1**: Please include a complete copy of PLOS’ questionnaire on inclusivity in global research in your revised manuscript.

**Response**: Completed questionnaire included with revised manuscript.

**Comment 2**: Our staff editors have determined that your manuscript is likely within the scope of our Diagnostics in Global Health Call for Papers.

**Response**: Acknowledged.

**Comment 3**: Please send a completed 'Competing Interests' statement, including any COIs declared by your co-authors. If you have no competing interests to declare, please state "The authors have declared that no competing interests exist". Otherwise please declare all competing interests beginning with the statement "I have read the journal's policy and the authors of this manuscript have the following competing interests:"

**Response**: Updated in submission system and revised statement added to the Declarations page.

**Comment 4**: Please amend your detailed Financial Disclosure statement. This is published with the article. It must therefore be completed in full sentences and contain the exact wording you wish to be published.

**Response**: Updated in submission system and revised statement added to the Declarations page.

**Comment 5:** Please provide separate figure files in .tif or .eps format only and remove any figures embedded in your manuscript file. Please also ensure that all files are under our size limit of 10MB.

**Response**: All figures converted to .tif and uploaded.

**Comment 6:** Figure 1: please (a) provide a direct link to the base layer of the map (i.e., the country or region border shape) and ensure this is also included in the figure legend; and (b) provide a link to the terms of use / license information for the base layer image or shapefile.

**Response**: Information added to Figure 1 legend.

**Comment 7:** Figure 3 includes an image of an identifiable person. Please provide written confirmation or release forms, signed by the subject(s) (or their parent/legally authorized guardian), giving permission to be photographed and to have their images published under our CC-BY 4.0 license.

**Response**: Signed release forms attached.

**Comment 8:** We have noticed that you have uploaded Supporting Information files, but you have not included a list of legends. Please add a full list of legends for your Supporting Information files after the references list.

**Response**: Information added.

**Reviewer #1**

**Comment 1**: The title requires minor edits

**Response**: We presume this comment refers to the comments as noted in the attached pdf document submitted by Reviewer 1, which states, “Is it possible to add patient monitoring and surveillance for HIV somewhere in the title?” Given that the study examines the feasibility of the test itself, (e.g., HIV-1 viral load testing) rather than specific uses of the (i.e., patient monitoring and surveillance), we prefer to leave the original title.

**Comment 2**: The background of the study in not clearly described as to why it is relevant - what was happening the BHC to warrant this pilot study? That is missing out

**Response**: The history of VL testing in Uganda is described in the second paragraph, starting on **Line** **77**. Specifically, we highlight that lower-level health facilities do not have the resources to perform this testing on site. We then highlight potential drawbacks of a centralized approach. In the subsequent paragraphs, we give a brief history of implementation of PoC testing for both TB and HIV, while again highlighting that similar efforts have not taken place at lower-level health facilities, which leads directly to our objectives and hypotheses, starting on **Line 110**.

In response to the Reviewer’s comments, we have added further description to the Study Site, starting on **Line** **132**, to clarify that BHC – like all other Level III Health Centers in Uganda – does not normally have PoC testing for HIV VL. The relevant sentence now reads:

*However, as a Level III Health Center, BHC does not have on-site HIV VL testing. Instead, dried blood spots (DBS) are collected and sent by motorcycle to the nearest hub site, approximately 25 km away, from where they are forwarded on to UNHLS.*

**Comment 3**: Phase should have incorporated pre-COVID-19 UNHLS data so that the pandemic impact would be well elucidated

**Response**: The study was developed prior to the emergence of the COVID-19 pandemic as indicated in our ClinicalTrials.gov registration. As such, the two pre-specified phases have nothing to do with COVID-19. We note in the Discussion section (Line 362) that the pandemic potentially impacted clinic attendance in Phase 2, but this was unrelated to any study design element or outcome.

**Comment 4**: I have attached comments on the PDF document which can help your team of authors to polish the document

**Response**: We have revised the manuscript with these suggestions. Minor changes to correct grammatical errors or improve readability were made throughout. Responses and revisions to more specific comments are documented below:

Abstract: Revised to incorporate the following: “I miss the importance of strengthening monitoring and surveillance of HIV patient clinical outcomes to reach HIV epidemic control targets.”

Abstract: Incorporated, “After assay, add: conducted at rural primary health center”

Background: Expanded description of MTB/RIF testing network, including reference, (**Line 101**), in response to “How is the current placement of GeneXpert MTB/RIF network within Uganda and specifically in focus to the study site where you conducted the pilot for HIV VL monitoring?”

Study Site: In response to, “Can reading audience be informed how the authors are associated with the Bugoye sub-county? Why this sub-county and not others even next door?” we believe that detailed history of our partnership at the site is beyond the scope of this manuscript.

Methods: In response to, “As part of methods, where is permission sought from health facility to access patient records and were patients informed about this activity to get their consent?” we note that this activity is covered under the local IRB review as noted in the Ethical Approvals section (**Line 247**). Specifically, we note that the MUST REC requires a letter of support from the local District Health Office before research can be approved. All participants were made of the study procedures outlined as is required by international regulations. Therefore, we do not believe that standard human subjects research methods need to be reported in the manuscript beyond the approvals of the review committees.

Methods: In response to, “Was NPoC VL test result approved for issuance by MoH Uganda? UNHLS VL test result should be the legitimate result per protocol, right?” we have clarified (**Line 163**) that the UNHLS results, which were performed in parallel remained the standard for clinical decision making.

Results” In regard to, “I expected phase 1 (3 months) to include 6 months before so that two time periods would be comparable as there would be pre-COVID-19 data on VL versus COVID-19 data on VL. Wouldn't this data be routinely available at BHC?” Please see our response to Comment 3. In brief, the phases had no relation to the COVID-19 pandemic.

**Comment 5**: Update the discussion session with the highlights flagged out in the results section

**Response**: See responses above to Comment 4.

**Comment 6**: Make the conclusion concise based on the results and discussion - looking the title can help firm up your conclusion

**Response**: Please see revised conclusion, which now reads:

*Implementation of a rapid, near point-of-care VL assay at a lower-level health center in rural Uganda appears feasible, but interventions to promote rapid clinical response and influence patient preferences about result receipt require further study. Given further evidence from higher-level facilities suggesting that implementation could be a key strategy to improving rates of VLS and achieving the UNAIDS targets, additional efforts are needed to expedite decentralized testing.*

**Reviewer #2**

**General Comments:** This is a rather interesting study on the opportunity for using Xpert for POC VL monitoring. It contributes to the current body of knowledge on the feasibility and acceptability of POC monitoring. Though, there are some major points where I feel improvement is warranted.

**Response**: Thank you. No specific changes made to manuscript.

**Comment 1**: The rationale of the study is not clear to me. I believe there is quite some literature showing feasibility already

**Response**: The reviewer is correct that there is increasingly robust evidence demonstrating the feasibility PoC VL testing for HIV in Uganda. We reference a number of these studies in our manuscript. However, our study is unique – and thus contributes to the literature – in that it was conducted at a level III health center, as opposed to higher-level facility such as a district hospital, referral hospital, or PEPFAR-funded clinic.

In response to this comment, we have further highlighted the lower-level facility throughout the text including:

- Abstract (Lines 45, 53)
- Background (Line 116)
- Methods (Line 139)
- Discussion (Lines 317, 371)

**Comment 2**: This project is called a pilot-feasibility study, though I think that is the wrong terminology. Pilot-feasibility studies are studies done to establish whether a clinical trial can be done. In such a case, you look at a number of factors such as being able to obtain enough participants? Number of times an intervention was used? Are all procedures for the trial in place? I would rather call this kind of an 'assessment to establish feasibility of POC monitoring in a rural cinic....' unless it was done in preparation of a trial, which would be mostly interesting, but then that should be described as the rationale

**Response**: We have added “to inform future trials” to the study objectivevs (Line 117)

**Comment 3**: I feel more information is needed on the validity with the used sample size. The size is small and it is questionable to related results

**Response**: There is greater recognition that sample size calculation should not be utilized in pilot feasibility studies. In response to this comment, we have clarified our rationale and provided relevant references. The relevant revision (Line 248) now reads:

*Due to the pilot nature of the study, no power calculations were conducted for the primary or secondary outcomes [26, 27].*

**Comment 4**: The specific objectives or secondary objectives in the introduction are not clear. The overarching objective is described would (I believe) to a qualitative design including observation and interviews with health care workers. Though a totally different methodology is used which I believe does not answer the overarching objective. It does answer the primary and secondary objectives though, so I believe the overarching objective should be adapted.

**Response**: It is unclear if the Reviewer is referencing the overarching objective (**Line 114**), for which there is only one overarching objective as opposed to the primary and secondary outcomes, which are described both in the Methods section (**Line 156)** as well as at ClinicalTrials.gov. We note that the outcomes of interest, both quantitative and qualitative, determined the methods, which informed our assessment of the overarching objective. Therefore, we believe the current organization is appropriate.

**Comment 5:** The objectives mentioned on page 6 are very smart and understandable. But I do not see how they will answer to the overarching objective stated at the end of the introduction on page 4.

**Response**: Again, the Reviewer seems to be confusing the terms objectives and outcomes. Please see our response to Comment 4. In brief, we would assert that outcome measures such as the number of tests performed per day inform our assessment of the overarching objective, namely determining if the requisite systems and process are in place.

**Comment 6:** How were the measures of feasibility chosen? If it is about feasibility of conducting a study, that should be stated more explicitly. I believe the mentioned measures are not so much about feasibility but an outcome of the performance of the test and procedures

**Response**: Using the framework outlined by Proctor et al (now Ref #21), we selected secondary outcome measures that would be required to achieve the potential benefit of NPoC VL testing. We have clarified this sentence, (Line 158), which now reads:

*Secondary outcomes included additional domains of feasibility that would be required to realize the potential of NPoC VL testing [21] including (i) the number of days between sample collection and the result returning to the clinic (i.e., the time-to-result), and (ii), number of days between sample collection and the patient receipt of the result (i.e., time-to-patient).*

**Comment 7:** Line 149: How was the measure of acceptability chosen? To me 'electing results by waiting' is not directly a measure of acceptability. There are many theoretical models for both acceptability and feasibility that could have been used. If such theories are not used, it should be clearly discussed why not and why the chosen measures were used

**Response**: We further evaluated acceptability through semi-structured interviews and focus group discussions, which will be reported separately. We have added a sentence (**Line 163**) to clarify this, which reads:

*Further evaluation of acceptability was elicited through participant interviews and focus-group discussions, which will be reported separately.*

**Comment 8:** More details are needed about the amount of blood collected

**Response**: We have clarified the amount of blood (~5 ml) in **Line 191.**

**Comment 9:** Table 1: Why do you display both the mean and the median?

**Response**: This is a common statistical practice when skew may be present. We also believe it is valuable to see the range of participant ages.

**Comment 10:** Line 299: I do not see how this conclusion can be drawn from the results. The results do not say anything about modest infrastructure improvement and staff training. This was not measured

**Response**: The infrastructure referred to the installation of an air conditioning unit (**Line 227**). However, we did not measure performance with and without the unit. We have removed this throughout.

**Comment 11:** More discussion is needed on possible implementation strategies to decrease time-to-patient for POC

**Response**: In response to the Reviewer’s comment, we have added the following (**Line 354**):

*One relatively simply intervention may be rearranging clinic workflows, which currently relegate phlebotomy and laboratory testing as the final step before discharge. With prior review of scheduled attendees, patients due for VL testing could have blood drawn on arrival, thereby reducing potential wait times.*

**Comment 12:** Discussion is needed on how the study can contribute to the current WHO guidelines which state that enhanced adherence counseling should be done at 50 copies (previously 1000 copies). In addition, I have been informed that most East African countries currently move to a different threshold varying from 50-200 copies, so this should be discussed as the study is of high relevance for that.

**Response**: We believe that this is beyond the scope of the current manuscript as Uganda has not yet (to our knowledge) changed the VL thresholds.

**Comment 13:** Please, adapt title and abstract based on the above points as well

**Response**: Changes made consistent with responses to above comments.

All authors have seen and approved the manuscript, contributed significantly to the work. The material presented here has not been published in any format and it is not being considered for publication elsewhere. We hope that this manuscript of substantial interest to your readers*.* Thank you for your time and consideration.

Kind regards,

Ross M. Boyce MD, MSc

Assistant Professor

Division of Infectious Diseases
